# Thrombo-inflammation as a missing variable in thoracic aortic intervention timing: toward a biology-informed threshold

**DOI:** 10.3389/fcvm.2026.1910157

**Published:** 2026-08-04

**Authors:** Vasiliki Androutsopoulou, Serge Sicouri, Prokopis-Andreas Zotos, Basel Ramlawi, Thanos Athanasiou, Dimitrios E. Magouliotis

**Affiliations:** 1 Department of Cardiothoracic Surgery, Faculty of Medicine, University of Thessaly, Biopolis, Larissa, Greece; 2 Department of Cardiac Surgery Research, Lankenau Institute for Medical Research, Wynnewood, PA, United States; 3 Department of Cardiac Surgery, Lankenau Medical Center, Wynnewood, PA, United States; 4 Department of Surgery and Cancer, Imperial College London, London, United Kingdom

**Keywords:** aortic aneurysm, aortic dissection, neutrophil extracellular traps, thrombo-inflammation, vascular remodeling

## Abstract

Aortic dissection and aneurysm have long been framed as mechanical failures of a degenerating wall. A growing body of evidence suggests that they may also reflect a shared thrombo-inflammatory process, in which coagulation and innate immunity are increasingly proposed to contribute actively to wall destruction rather than merely to indicate it. This review synthesizes that shift across the dissection and aneurysm spectrum. We first delineate the core machinery, including tissue-factor-driven coagulation, platelet and leukocyte interactions, neutrophil extracellular traps, inflammasome signaling, and complement activation. We, then connect these mediators to the proteolytic and phenotypic events that remodel the aortic wall: matrix metalloproteinase activation, elastin and collagen degradation, smooth muscle cell phenotype switching, apoptosis, and ferroptosis. We contrast disease-specific biology, the acute thrombo-inflammatory storm and false-lumen dynamics of dissection against the intraluminal thrombus and complement consumption that characterize aneurysm. A dedicated section examines the endothelial interface on which these processes depend, integrating junctional integrity, membrane-channel function, and cyclic-nucleotide microdomain regulation as the permissive substrate linking a compromised barrier to leukocyte engagement and maladaptive remodeling. We evaluate candidate circulating biomarkers, reinterpret endovascular therapy through a thrombo-inflammatory lens by addressing post-implantation inflammatory responses and false-lumen thrombosis, and outline how multi-omics and computational integration of the inflammatory, remodeling, and endothelial axis may refine preoperative risk assessment. The aim of our study is a mechanistically coherent framework connecting molecular drivers to clinical strategy.

## 1 Introduction

Aortic dissection and aortic aneurysm sit at opposite ends of a single degenerative continuum, yet they share a common and lethal endpoint: catastrophic wall failure. Contemporary management remains anchored to anatomic surrogates, principally maximal diameter and growth rate, with operative thresholds and surveillance intervals codified by recent consensus guidance (1). These metrics are pragmatic and have served the field well, however they are imperfect predictors. A substantial fraction of acute dissections and ruptures occur in aortas below the diameter at which intervention would be recommended, exposing a gap between the geometry we measure and the biology that determines when a wall actually gives way.

That gap has motivated a conceptual shift. The aortic wall is increasingly understood not as an inert mechanical structure that simply fatigues under pulsatile load, but as a living tissue whose failure is precipitated by an active biological program. Central to that program is thrombo-inflammation: the tightly coupled engagement of the coagulation cascade and innate immunity at the vessel wall. In this framework, thrombin generation, platelet activation, neutrophil and monocyte recruitment, and complement deposition are proposed to act not as mere epiphenomena of injury but as candidate mechanistic drivers of proteolysis, smooth muscle cell loss, and medial degeneration, a hypothesis that the balance of current evidence supports but has not yet definitively established.

This review synthesizes the thrombo-inflammatory model across dissection and aneurysm. We first set out the core mediators, then trace how they remodel the wall, before contrasting disease-specific biology. A dedicated section of the manuscript addresses the endothelial interface, the barrier on which the thrombo-inflammatory cascade depends, integrating junctional, channel, and cyclic-nucleotide regulation to define the permissive substrate of disease. We then consider biomarkers, reinterpret endovascular therapy through this lens, and close with priorities for multi-omics and computational integration. Given the heterogeneity of the underlying literature, including murine models, human tissue studies, and clinical cohorts of varying design, we adopt a narrative rather than systematic review, to integrate mechanistic and translational evidence into a coherent conceptual framework.

## 2 The thrombo-inflammatory machinery

**Figure 1:**
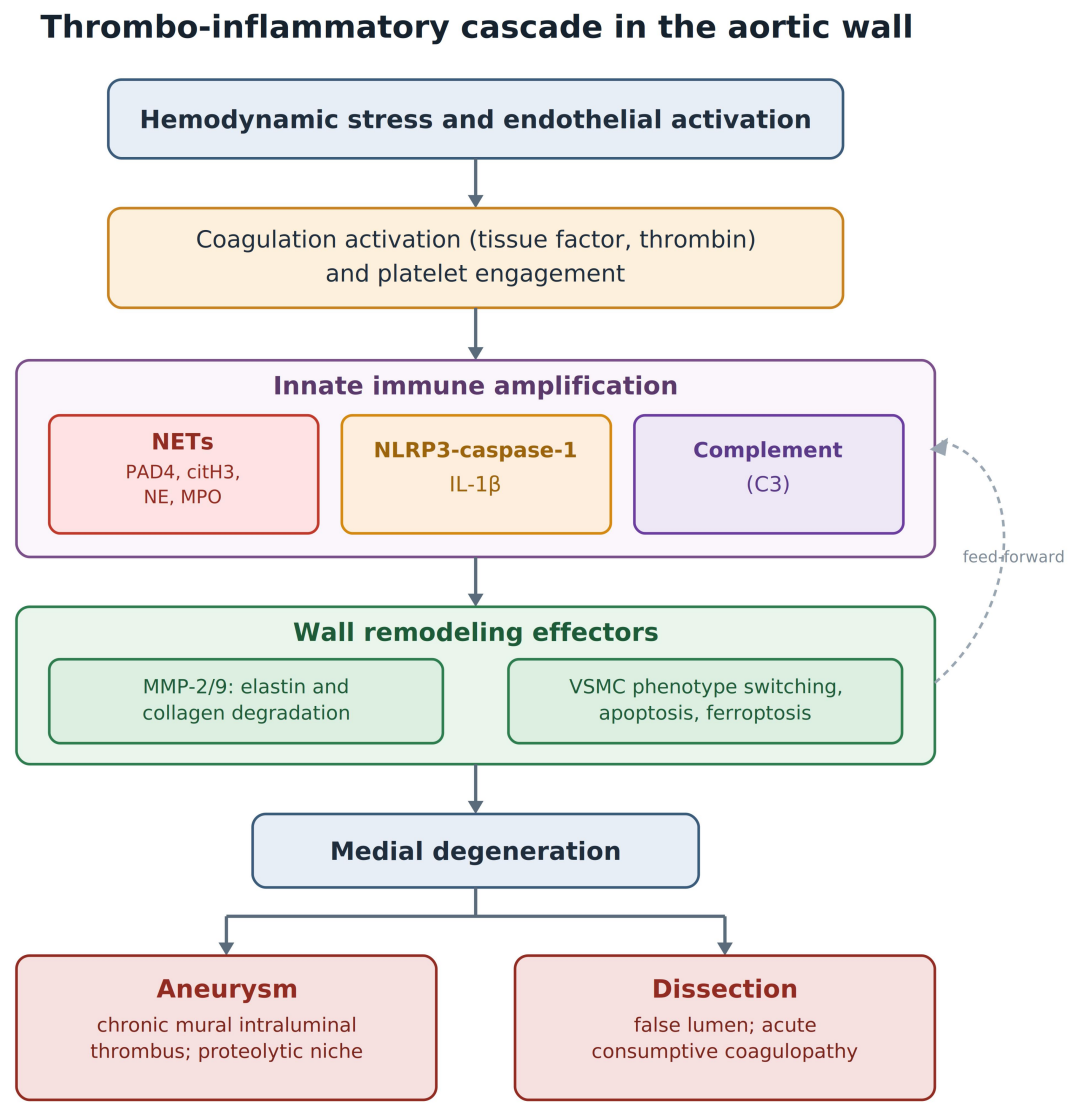
The thrombo-inflammatory cascade driving aortic wall failure. Hemodynamic stress and endothelial activation initiate coagulation and platelet engagement, which amplify through neutrophil extracellular traps, the NLRP3-caspase-1 axis, and complement. The resulting effectors degrade the extracellular matrix and drive vascular smooth muscle cell dysfunction, producing medial degeneration that manifests as aneurysm or dissection. citH3, citrullinated histone H3; MMP, matrix metalloproteinase; MPO, myeloperoxidase; NE, neutrophil elastase; NETs, neutrophil extracellular traps; PAD4, peptidylarginine deiminase 4; VSMC, vascular smooth muscle cell.

Thrombo-inflammation refers to the reciprocal amplification between hemostasis and innate immunity. Endothelial activation and exposure of tissue factor initiate thrombin generation and platelet activation, while activated platelets and the developing fibrin scaffold in turn recruit and prime leukocytes. The result is a self-reinforcing loop in which clotting promotes inflammation and inflammation promotes clotting, localized to the aortic wall and its luminal surface (Figure 1).

Neutrophil extracellular traps (NETs) have emerged as a pivotal node in this loop. Through peptidylarginine deiminase 4 (PAD4)-dependent histone citrullination, neutrophils extrude decondensed chromatin studded with neutrophil elastase (NE), myeloperoxidase, and cell-free DNA. Therefore, they generate a procoagulant, cytotoxic, and proteolytic mesh. In aortic dissection, integrated multi-omics profiling has shown that phenotype-associated macrophages orchestrate NET formation through the CXCL3 to CXCR2 axis. This increased NET formation is a defining feature of both the systemic immune state and the local aortic microenvironment, by inhibiting NET formationblocking citrullinated histone H3 or the CXCL3 to CXCR2 axis, attenuates progression and rupture of aortic aneurysms in mice; notably, plasma citrullinated histone H3 predicted adverse aortic events after endovascular therapy (2). In abdominal aortic aneurysm, NETs contribute to wall degradation through direct cytotoxicity to vascular smooth muscle cells, induction of phenotypic switching and ferroptosis, and amplification of inflammation, with PAD4, NE, and myeloperoxidase among the key effectors (3). Genetic and pharmacologic studies place neutrophil elastase causally upstream of dissection: NE expression and plasma activity rise in experimental and human thoracic aortic dissection, and NE deletion reduces NET formation in the dissected wall (4).

The NLRP3 inflammasome links danger sensing to the maturation of interleukin-1 beta (IL-1 beta) and provides a second mechanistic axis. In human sporadic thoracic aortic aneurysm and dissection, smooth muscle contractile protein degradation is associated with activation of the NLRP3 and caspase-1 cascade, and caspase-1 can directly cleave contractile proteins, producing contractile dysfunction (5). Pharmacologic NLRP3 inhibition with MCC950 reduces the incidence of dissection and protects the wall in murine models (6). Moreover, IL-1 beta itself upregulates endothelial adhesion molecules, promotes monocyte recruitment, and stimulates smooth muscle cells to proliferate and to release IL-6 and monocyte chemoattractant protein-1, therefore propagating inflammation (7). Necrotic cell debris can prime the inflammasome in aneurysm-derived smooth muscle cells, releasing senescence-associated IL-6 and MCP-1 (8).

Complement activation completes the innate-immune triad. Proteomic interrogation of the intraluminal thrombus in human abdominal aortic aneurysm has highlighted retention, consumption, and proteolysis of complement within the thrombus, with reduced systemic C3 in larger aneurysms, a state that promotes neutrophil chemotaxis and a detrimental local milieu (9). Together with platelet and leukocyte aggregates and macrophage polarization, these mediators constitute a coordinated thrombo-inflammatory apparatus poised to remodel the wall. Table 1 summarizes the principal mediators and their actions across dissection and aneurysm.

**Table 1:** Core thrombo-inflammatory mediators in aortic dissection and aneurysm.

Mediator or axis	Principal actions in the aortic wall	Predominant context	Ref.
NETs (PAD4, citH3, NE, MPO)	Chromatin extrusion; VSMC cytotoxicity, phenotype switching and ferroptosis; procoagulant and proteolytic mesh	Dissection and AAA	(2–4)
NLRP3-caspase-1 and IL-1 beta	Caspase-1 cleaves SMC contractile proteins; IL-1 beta drives endothelial adhesion molecules, IL-6 and MCP-1	Dissection and aneurysm	(5–8)
Complement (C3)	Retention, consumption and proteolysis within thrombus; neutrophil chemotaxis	AAA (intraluminal thrombus)	(9)
Coagulation and platelets (tissue factor, thrombin)	Thrombin signaling, platelet-leukocyte aggregates, consumptive coagulopathy	Acute dissection	(14)
Matrix metalloproteinases (MMP-2/9)	Elastin and collagen degradation; progressive wall weakening	Dissection and aneurysm	(3, 12)
Intraluminal thrombus (neutrophil-rich)	Oxidative and proteolytic niche; hypoxia and inflammatory infiltration; correlates with growth	AAA	(9, 12, 13)

AAA, abdominal aortic aneurysm; citH3, citrullinated histone H3; IL, interleukin; MCP-1, monocyte chemoattractant protein-1; MMP, matrix metalloproteinase; NE, neutrophil elastase; NETs, neutrophil extracellular traps; PAD4, peptidylarginine deiminase 4; SMC, smooth muscle cell; VSMC, vascular smooth muscle cell.

Underlying these individual mediators is a single organizing principle: immunothrombosis, the physiological coupling of coagulation and innate immunity that becomes maladaptive when sustained (10). Platelets are central to this coupling. Beyond aggregation, activated platelets present P-selectin and CD40 ligand, form heterotypic aggregates with neutrophils and monocytes, and lower the threshold for NET release, while the developing thrombus concentrates thrombin and chemokines that further recruit leukocytes. Von Willebrand factor reinforces the loop under the high wall-shear conditions of the aorta: ultra-large multimers unfold to capture platelets and to bind NET-derived DNA, building a reactive scaffold for leukocyte adhesion at the injured surface (10). The terminal complement pathway converges on the same surface, where C5a amplifies neutrophil and monocyte chemotaxis and the membrane attack complex injures endothelium and smooth muscle. Read together, these interactions explain why the aortic wall does not experience NETs, inflammasome activation, and complement as separate insults but as one integrated thrombo-inflammatory program, each arm potentiating the others. This integrated perspective also reframes wall vulnerability as a function of the degree to which these loops are engaged, rather than merely the extent of luminal dilation.

Clinically, the value of this machinery lies less in any single mediator than in the prospect that its circulating footprint might one day flag a wall in which these loops are actively engaged; plasma citrullinated histone H3, for example, has already shown prognostic value after endovascular therapy (2), and other candidate analytes such as D-dimer and the neutrophil-to-lymphocyte ratio are examined in detail in Sec. 6. At present these associations are diagnostic and prognostic rather than validated triggers for intervention, and the open translational question is whether they can be developed into a reproducible readout of biological activity that meaningfully complements diameter.

## 3 Thrombo-inflammation as a remodeling engine

The destructive output of this apparatus is vascular remodeling. The hallmark is degradation of the load-bearing extracellular matrix. Matrix metalloproteinases, principally MMP-2 and MMP-9, together with neutrophil-derived proteases and a plasmin-generating environment, cleave elastin and collagen, the structural proteins that confer the aorta its resilience and recoil. Elastin fragmentation and disorganized collagen turnover progressively reduce the wall's capacity to bear pulsatile stress.

In parallel, the resident vascular smooth muscle cell population is depleted and dysfunctional. Under thrombo-inflammatory pressure, smooth muscle cells switch from a contractile to a synthetic phenotype, lose their characteristic contractile markers, and become susceptible to apoptosis and to ferroptosis, an iron-dependent form of regulated cell death implicated downstream of NET exposure (3). The NLRP3 and caspase-1 axis contributes directly by degrading contractile proteins (5), while necrotic debris sustains a feed-forward inflammasome response within the aneurysmal media (8). The combined loss of contractile competence and structural matrix yield medial degeneration, the histologic substrate common to aneurysmal dilation and to the cleavage planes that propagate dissection. Figure 1 integrates this cascade from initiating stimulus to clinical phenotype.

Mononuclear phagocytes determine whether this remodeling is contained or propagated. Infiltrating monocytes differentiate along a spectrum conventionally framed as classically activated (M1) and alternatively activated (M2) macrophages: the former secrete IL-1 beta, IL-6, tumor necrosis factor, and matrix metalloproteinases that drive proteolysis, whereas the latter release IL-10 and transforming growth factor beta and support matrix repair (11). The balance between these programs, together with the efficiency of efferocytosis, the clearance of apoptotic cells that normally resolves inflammation, shifts during disease progression and differs between the intraluminal thrombus, media, and adventitia, so that the same lineage can either restrain or accelerate wall destruction depending on context (11). Impaired efferocytosis leaves a growing burden of secondary necrotic debris that, as noted, re-primes the inflammasome (8). Sustained inflammation also drives adventitial neovascularization from the vasa vasorum, delivering further inflammatory cells and creating fragile microvessels prone to intramural hemorrhage. Macrophage polarization thus operates as a rheostat on the remodeling engine, linking the molecular mediators of Sec. 2 to the tissue-level outcome.

For the clinician, the remodeling engine reframes what a stable diameter may conceal, since a quiescent wall and a wall undergoing active proteolytic turnover can appear identical on cross-sectional imaging. Circulating markers of matrix turnover, such as MMP-9 and elastin degradation products, are attractive precisely because they report on this proteolytic activity (3), although none has yet been prospectively validated as a determinant of surveillance interval or operative timing.

## 4 Disease-specific biology

Although they share keys mediators, aneurysm and dissection manifest thrombo-inflammation through distinct anatomic substrates. In abdominal aortic aneurysm, the intraluminal thrombus is a defining and biologically active feature rather than an inert deposit. Most aneurysms large enough to warrant repair contain such thrombus, and its presence reorganizes the biology of the underlying wall: neutrophils accumulate within the thrombus and create an oxidative and proteolytic environment, thrombus thickness correlates with aneurysm diameter, matrix metalloproteinase levels, elastin degradation, and smooth muscle cell apoptosis The resulting localized hypoxia drives adventitial angiogenesis and inflammatory infiltration (12). The thrombus may relieve peak wall stress mechanically, yet its proteolytic weakening of the covered wall appears to predominate, and larger thrombus burden is associated with faster growth (12, 13). Complement consumption within the thrombus reinforces this detrimental role (9). Thoracic aortic aneurysm, by contrast, is characterized more by medial degeneration than by luminal thrombosis, with additional biological distinctions between bicuspid and tricuspid aortic valve phenotypes.

Acute aortic dissection represents the most fulminant expression of the thrombo-inflammatory process. The entry tear and the resulting false lumen create an expansive thrombogenic surface that drives intense local and systemic activation of coagulation and innate immunity. Clinically, this manifests as a consumptive coagulopathy: a scoping review of disseminated intravascular coagulation in aortic disease found that disseminated intravascular coagulation occurs in roughly one in five dissections and is significantly related to false-lumen diameter and length. It is associated with markedly higher in-hospital mortality, and concurrent thrombocytopenia and elevated D-dimer identify a particularly high-risk subgroup with substantially increased mortality (14). Neutrophil elastase and NETs are causally implicated in the dissecting wall (4), and false-lumen thrombosis status is a key determinant of downstream remodeling. The contrast between aneurysm and dissection is instructive: in aneurysm the thrombus is chronic, mural, and slowly corrosive, whereas in dissection the thrombo-inflammatory burden is acute, luminal, and systemically consumptive.

Anatomic subtype further refines this picture. In acute type A dissection, the thrombo-inflammatory storm is most intense and most systemic, reflecting the proximal entry tear, the large false-lumen surface, and frequent pericardial and coronary involvement, whereas in type B dissection the burden is more regional and the degree of false-lumen thrombosis becomes the principal determinant of subsequent remodeling (14). Thoracic aneurysm biology is similarly heterogeneous: bicuspid aortic valve aortopathy follows a molecular trajectory distinct from that of tricuspid-associated and sporadic disease, with differences in smooth muscle phenotype, matrix turnover, and the epigenetic programs that regulate them (15). These distinctions matter for a thrombo-inflammatory framework because they predict where the cascade will be most active and which patients carry the greatest latent risk below conventional size thresholds, reinforcing the rationale for biology-informed rather than purely diameter-driven stratification (2).

The practical implication of this disease-specific biology is that a single biological readout is unlikely to serve aneurysm and dissection equally, and that any biology-informed strategy will need to be calibrated to disease phase and anatomic subtype (14, 15) rather than applied uniformly across the aortic organ.

## 5 The endothelial interface

**Figure 2:**
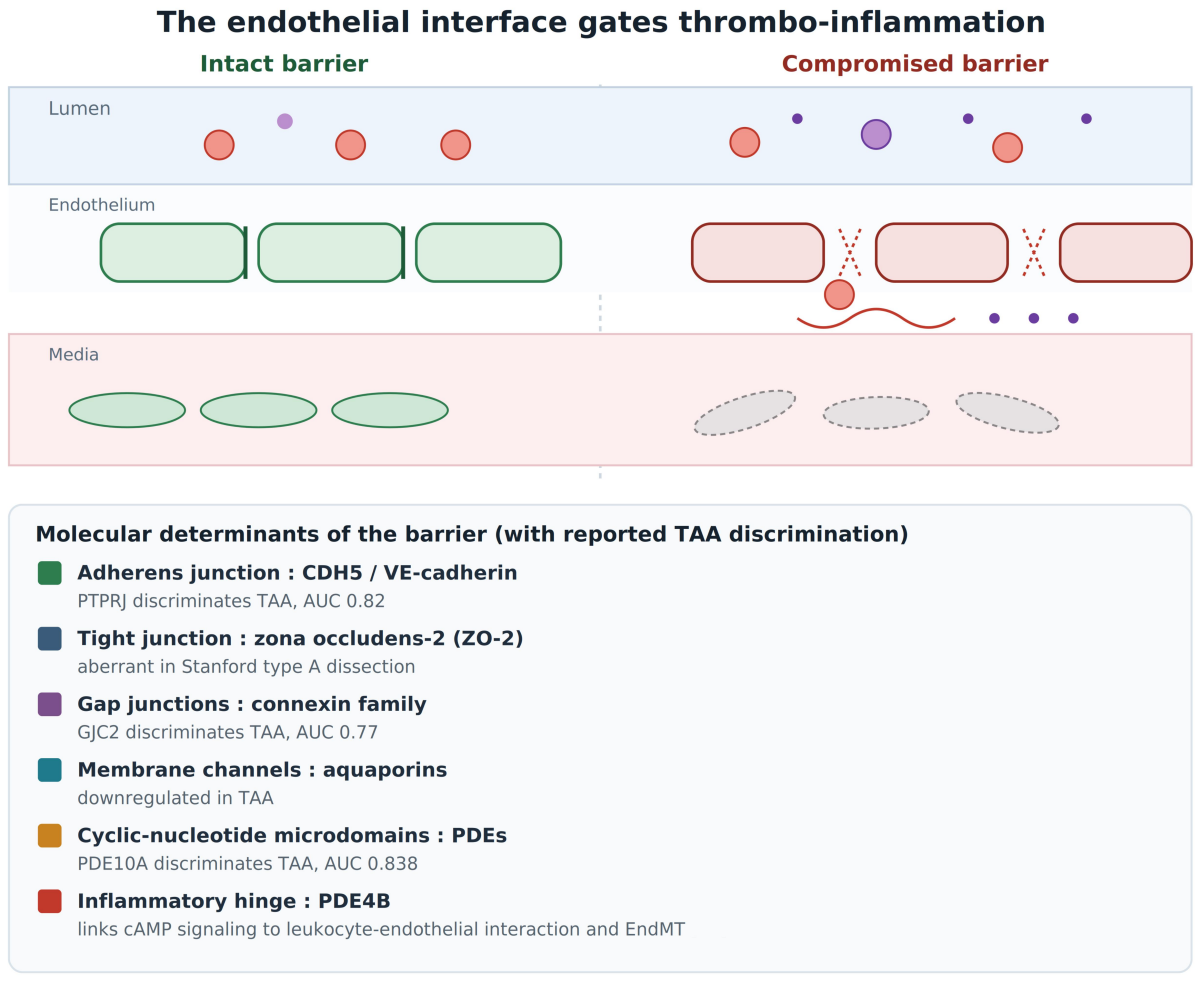
The endothelial interface as the permissive substrate of thrombo-inflammation. An intact intimal barrier (left) resists leukocyte transmigration, NET deposition, and complement access, whereas a junctionally and signalling-compromised barrier (right) permits them, exposing the media to remodeling. The legend maps each barrier component to its reported discriminatory performance for thoracic aortic aneurysm. AUC, area under the receiver-operating-characteristic curve; CDH5, cadherin-5; EndMT, endothelial-to-mesenchymal transition; NET, neutrophil extracellular trap; PDE, phosphodiesterase; TAA, thoracic aortic aneurysm; VE-cadherin, vascular endothelial cadherin.

Thrombo-inflammation does not act on an indifferent surface. Every step of the cascade is gated by the state of the intimal endothelium and its intercellular junctions. The endothelial interface therefore functions as the permissive substrate that determines whether a thrombo-inflammatory stimulus is contained or converted into wall failure. Several converging lines of evidence define this substrate at the molecular level (Figure 2).

Junctional integrity is the first determinant. Adherens junctions, organized around cadherin-5 (vascular endothelial cadherin), maintain barrier cohesion; an in-depth analysis of the cadherin-5 interactome in thoracic aortic aneurysm identified an eight-gene endothelial signature, within which protein tyrosine phosphatase receptor type J discriminated aneurysmal from control aorta with excellent performance (area under the curve 0.82) (16). Tight junctions are likewise implicated: zona occludens-2 is aberrantly expressed in Stanford type A aortic dissection, consistent with a loss of barrier control in the acute setting (17). Gap junctions complete the junctional picture, with eight gap junction proteins downregulated in aneurysmal aorta and four (including GJC2, area under the curve 0.77) showing fair discriminatory ability, implicating impaired intercellular communication in pathogenesis (18).

Beyond cell-cell junctions, membrane transport and intracellular signaling shape barrier behavior. Aquaporin water channels are downregulated in thoracic aortic aneurysm, supporting a broader model in which transport channels and junctional complexes co-determine aortic integrity (19). Cyclic-nucleotide microdomains provide a further regulatory layer: a family-wide transcriptomic analysis of phosphodiesterases in thoracic aortic aneurysm found thirteen dysregulated isoforms, with PDE10A emerging as a central signature (area under the curve 0.838), and a pattern of cyclic-nucleotide rewiring predicted to favor endothelial barrier fragility and maladaptive smooth muscle remodeling (20). Of particular relevance to thrombo-inflammation, PDE4B sits directly at the interface of cyclic-AMP signaling and leukocyte-endothelial interaction and endothelial-to-mesenchymal transition, offering a concrete molecular hinge by which microdomain dysregulation could license inflammatory engagement of the wall (20). These junctional, channel, and cyclic-nucleotide programs are further coordinated by reversible epigenetic mechanisms, DNA methylation, histone modification, and microRNA regulation, that govern endothelial permeability, smooth muscle phenotype, and matrix turnover in aortic disease (15).

Synthesized, these data describe a permissive endothelial state: a junction-poor, channel-depleted, cyclic-nucleotide-dysregulated intima that loses barrier control and thereby allows the thrombo-inflammatory mediators of Secs. 2 and 3 to engage and remodel the wall. This molecular picture mirrors the longitudinal biomechanical vulnerability observed clinically, reinforcing the view that barrier biology and wall mechanics are two readouts of the same underlying failure (21). Positioning the endothelial interface in this way supports a model in which thrombo-inflammation may be better understood not as an event imposed on a passive wall but as a process whose magnitude is shaped, at least in part, by the integrity of the surface it meets. This remains a mechanistic hypothesis derived largely from tissue-level association studies rather than an established causal sequence.

It should be emphasized that the endothelial signatures summarized in this section, including PTPRJ (16), GJC2 (18), aquaporins (19), and the phosphodiesterase isoforms (20), derive from exploratory, single-cohort transcriptomic and bioinformatic analyses of surgically resected tissue. They are discovery-stage observations that discriminate diseased from control aorta within their source cohorts; they have not been measured in peripheral blood, replicated across independent populations, or linked to prospective clinical outcomes. They are therefore best regarded as mechanistic and hypothesis-generating rather than as clinically actionable biomarkers, and the comparatively detailed treatment they receive here reflects their conceptual role in defining the permissive substrate, not a corresponding maturity of clinical evidence.

## 6 Biomarkers

The thrombo-inflammatory model yields a rational menu of circulating biomarkers. D-dimer, the fibrin degradation product, is the best established: it is markedly elevated in acute aortic dissection and performs well as a rule-out test at the conventional 500 nanograms per milliliter threshold (22), and meta-analysis supports its diagnostic utility while cautioning that elevation is not specific to dissection (23). Beyond diagnosis, D-dimer carries prognostic weight, with higher levels independently associated with major adverse events and mortality, and with the combination of thrombocytopenia and elevated D-dimer identifying particularly high-risk patients (14, 24). Markers of NET formation, notably circulating citrullinated histone H3, extend this panel and have shown prognostic value after endovascular therapy (2). Additional candidates, including the neutrophil-to-lymphocyte ratio, IL-6, fibrinogen, and platelet indices, reflect the same underlying biology. Table 2 summarizes candidate markers across both the circulating and tissue compartments.

**Table 2:** Candidate biomarkers across the thrombo-inflammatory and endothelial axes.

Biomarker	Class	Role and evidence	Source	Ref.
D-dimer	Coagulation and fibrinolysis	Diagnostic rule-out (about 500 ng/mL); prognostic for major adverse events and mortality	Circulating	(14, 22–24)
Citrullinated histone H3	NET marker	Tracks NET burden; prognostic after endovascular therapy	Circulating	(2)
NLR, IL-6, fibrinogen, platelet count	Systemic inflammation and hemostasis	Reflect overall thrombo-inflammatory burden	Circulating	(14, 24)
PTPRJ (CDH5 interactome)	Endothelial, junctional	Discriminates TAA, AUC 0.82	Tissue	(16)
GJC2 (gap junction)	Endothelial, junctional	Discriminates TAA, AUC 0.77	Tissue	(18)
PDE10A	Cyclic-nucleotide signaling	Discriminates TAA, AUC 0.838; candidate composite marker	Tissue	(20)
Aquaporins	Membrane channel	Downregulated in TAA	Tissue	(19)

AUC, area under the receiver-operating-characteristic curve; CDH5, cadherin-5; IL-6, interleukin-6; NET, neutrophil extracellular trap; NLR, neutrophil-to-lymphocyte ratio; TAA, thoracic aortic aneurysm.

Systemic inflammatory and hematologic indices extend this circulating panel and are attractive precisely because they are inexpensive and universally available. The neutrophil-to-lymphocyte ratio integrates the innate-immune activation and relative lymphopenia that accompany acute aortic injury, and a systematic review and meta-analysis found that an elevated ratio is associated with increased mortality in aortic dissection, although heterogeneity across cohorts and the absence of a consensus cutoff temper its standalone use (25). The platelet-to-lymphocyte ratio, C-reactive protein, interleukin-6, and fibrinogen track the same thrombo-inflammatory burden and have been linked to disease severity and adverse events, while serial rather than single measurements appear more informative given the dynamic nature of the response. A second mechanistically grounded class reports on matrix turnover: circulating matrix metalloproteinase-9, tissue inhibitors of metalloproteinases, and elastin and collagen degradation products mirror the proteolytic activity that drives wall weakening, and several have correlated with aneurysm diameter and growth. Although none has yet achieved the diagnostic standing of D-dimer, each addresses a distinct node of the cascade and therefore represents a rational component of a multimarker strategy.

Nucleic-acid and vesicle-based markers represent a newer and rapidly expanding category. Circulating microRNAs, stabilized in plasma and serum through encapsulation within vesicles and protein complexes, are differentially expressed in aortic disease. Profiling studies in abdominal aortic aneurysm have highlighted species such as miR-155, linked to vascular inflammation (26), as well as the miR-29 family, whose downregulation promotes extracellular-matrix degradation in abdominal aortic aneurysm (27). Cell-free DNA, including the citrullinated-histone-bound DNA released during NET formation, offers a direct readout of the neutrophil arm of the cascade, and extracellular vesicles carry cargo that reflects the activation state of their parent endothelial, platelet, and leukocyte populations. These analytes are appealing because they link a circulating, repeatedly samplable signal to a specific biological mechanism, but assay standardization, normalization, and validation in adequately powered prospective cohorts remain prerequisites for clinical adoption.

The molecular signatures emerging from transcriptomic interrogation of the wall, including the endothelial and cyclic-nucleotide markers discussed above, represent a complementary class of candidate biomarkers (16, 18–20). Their translation is limited by the persistent gap between tissue-level discovery and detectability in peripheral blood; whether wall-derived signals are reliably reflected in circulation, or within circulating extracellular vesicles, remains the central question for their clinical development.

To state this plainly, the wall-derived transcriptomic markers cited here, including PDE10A (20), PTPRJ (16), and GJC2 (18), are exploratory findings from individual tissue cohorts and have not been shown to be detectable, let alone predictive, in the circulation. Describing them as candidate biomarkers denotes biological rationale and discovery-stage evidence only, and should not be read as an indication of clinical readiness.

A complementary route bypasses the blood entirely and interrogates the wall *in vivo* through molecular imaging. 18F-fluorodeoxyglucose positron emission tomography reports metabolic activity that localizes to macrophage-rich, inflamed segments, and focal uptake has been associated with symptomatic, rapidly expanding, and rupture-prone aneurysms, although a systematic review of imaging biomarkers concluded that its predictive value for growth and rupture is inconsistent across studies and not yet sufficient to guide individual decisions (28). Tracers aimed at more specific processes, such as 18F-sodium fluoride for microcalcification and active wall degeneration, and agents targeting macrophages or matrix proteases, are under investigation and may prove more discriminating. The broader lesson from both the circulating and imaging literatures is the same: no single marker, measured once, is likely to capture a process this distributed and dynamic. The most credible path is a composite that combines complementary readouts, for example a thrombo-inflammatory analyte, a matrix-turnover analyte, an endothelial or wall-derived signature, and an imaging index, integrated with clinical and anatomic data and validated prospectively against hard endpoints before it can refine the diameter-based thresholds that still govern practice (28). In practice, such a panel would also need to demonstrate incremental value beyond diameter and growth rate alone, and to remain robust across sexes and across the distinct biology of thoracic and abdominal disease, before it could responsibly enter routine surveillance.

The inconsistency of 18F-fluorodeoxyglucose positron emission tomography merits particular emphasis, because it illustrates the wider gap between biological plausibility and clinical utility. Reported associations between wall uptake and subsequent growth or rupture have not been reproducible across cohorts (28), an outcome attributable in part to heterogeneous acquisition protocols, the absence of standardized uptake thresholds, variable blood-pool correction, and small single-center samples with differing endpoints. As a result, metabolic imaging is not currently used to guide individual surveillance or operative decisions, and it stands as a cautionary example of how an intuitively attractive biomarker can fail to translate without prospective, standardized validation.

## 7 Endovascular strategies through a thrombo-inflammatory lens

Endovascular repair offers a clinical window onto thrombo-inflammation *in vivo*. Post-implantation syndrome, a sterile systemic inflammatory response marked by fever, leukocytosis, and elevated C-reactive protein, follows a substantial proportion of endovascular aortic repairs. Its reported incidence ranges from roughly one quarter to two fifths of cases [approximately 26% in one series (29) and up to 40% in another (30)], and it is attributed less to graft material *per se* than to endothelial disruption and the systemic response to rapid thrombus formation. Procedural factors, including the number of stent segments, contrast volume, operative time, and emergency status, further predict its occurrence (29, 30).

The clinical weight of this inflammatory response is debated but not negligible. Although post-implantation syndrome is usually self-limiting, it prolongs hospital stay and has, in some series, been associated with a higher rate of early major adverse events, so it is better regarded as a marker of host inflammatory activation than as an incidental fever. Its magnitude depends in part on the device itself: the fabric of the endograft is a consistent determinant, with woven polyester provoking a significantly stronger temperature and C-reactive protein response, than expanded polytetrafluoroethylene, an effect attributed to the differing capacity of these materials to activate circulating leukocytes (31). It should be noted that most comparative fabric data predate current-generation device designs and may not fully reflect contemporary practice. The volume of new-onset thrombus within the excluded sac and the extent of aortic coverage further modulate the response, consistent with the view that post-implantation syndrome reflects the combined burden of a foreign thrombogenic surface and the acute thrombus it nucleates (29, 30). Device selection and the anticipated thrombus load are therefore variables a thrombo-inflammatory framework would predict to matter, and that emerging evidence suggests do.

False-lumen thrombosis after thoracic endovascular repair illustrates the double-edged nature of thrombus in this setting. Complete false-lumen thrombosis is a therapeutic goal because it promotes positive aortic remodeling; adjunctive techniques such as candy-plug false-lumen embolization can achieve this, with reported true-lumen expansion and false-lumen regression (32). Yet the same thrombotic process is intrinsically thrombo-inflammatory, and the postoperative period is marked by measurable coagulation activation, with transient thrombocytopenia and elevated D-dimer that are more sustained in dissection, and overt postoperative disseminated intravascular coagulation in a non-trivial fraction of cases depending on the length of aortic coverage and the presence of endoleak (14). The prognostic value of plasma citrullinated histone H3 after endovascular therapy further situates NET biology within post-repair risk stratification (2). Anti-inflammatory prophylaxis, including perioperative glucocorticoids, has been explored to attenuate the post-implantation response, with evidence to date suggesting symptomatic benefit but no clear effect on hard outcomes, leaving the question open (33).

Beyond the acute phase, the excluded aneurysm sac remains a biologically active compartment. After endovascular repair, the sac typically thromboses, but the resulting thrombus is not inert: it sustains the same neutrophil-rich, proteolytic, and hypoxic microenvironment described for the native intraluminal thrombus, and ongoing wall inflammation may help explain why some sacs continue to enlarge even without a demonstrable endoleak (10, 11). Persistent perfusion through type II endoleaks maintains a cycle of clotting and lysis within the sac that can drive expansion, whereas durable thrombosis and sac regression mark a favorable, quiescent course. Viewed this way, sac behavior on surveillance imaging is in part a readout of whether the thrombo-inflammatory process has been extinguished or merely relocated, which reframes endoleak management as an effort to remove the residual stimulus rather than simply to seal a leak.

These observations argue for attention to the medical and surveillance environment around the procedure, not only the procedure itself. Statins, whose pleiotropic effects include suppression of vascular inflammation and stabilization of matrix metalloproteinase activity, are associated in meta-analysis with slower aneurysm growth, lower rupture rates, and reduced perioperative mortality, and their continuation through the perioperative period is consistent with the biology reviewed here (34). Antiplatelet and anticoagulant therapy must be balanced carefully, since the same thrombus that weakens the wall also contributes to favorable sac and false-lumen thrombosis, so blanket suppression of coagulation is not obviously beneficial and requires individualized judgment, the same caution that applies to systemic anti-inflammatory prophylaxis (33). Looking forward, the markers discussed in the preceding section offer a route to personalize surveillance: serial citrullinated histone H3 or other thrombo-inflammatory analytes (2), together with metabolic or wall-directed imaging (28), could in principle identify the patient whose excluded sac or treated dissection remains inflammatory and therefore at residual risk, shifting post-repair follow-up from fixed imaging intervals toward a biology-informed schedule. Equally, any anti-inflammatory or antithrombotic strategy adopted after repair will require the same prospective, endpoint-driven evidence demanded of the device itself, so that intensifying medical therapy translates into fewer reinterventions and ruptures rather than merely lower biomarker levels.

## 8 Therapeutic targeting of thrombo-inflammation

The thrombo-inflammatory model is attractive in part because its nodes are pharmacologically accessible, and several are already being tested in cardiovascular disease. The interleukin-1 beta axis has the strongest clinical precedent: in the CANTOS trial, the anti-IL-1 beta antibody canakinumab reduced recurrent major cardiovascular events in post-myocardial infarction patients with elevated high-sensitivity C-reactive protein independently of lipid lowering (35); no aortic-specific trial exists, but this result establishes proof of principle that sustained IL-1 beta signalling is a pharmacologically tractable driver of cardiovascular events, a logic that applies directly to the aortic thrombo-inflammatory cascade. For the aorta specifically, the inexpensive inflammasome-directed agent colchicine, which impairs neutrophil recruitment and NLRP3 assembly, has shown protection in experimental aneurysm and is under prospective evaluation for small abdominal aortic aneurysm in a randomized trial (36), complementing preclinical evidence that NLRP3 inhibition limits dissection (6). Whether these benefits translate to aortic growth and event reduction in patients remains to be established, but the rationale follows directly from the mediators reviewed above.

Other nodes are at earlier stages. NET-directed strategies, including PAD4 inhibition, DNase-mediated NET dissolution, and interruption of the macrophage CXCL3 to CXCR2 axis, attenuate dissection and aneurysm in animal models and are reinforced by the prognostic value of circulating NET markers after endovascular therapy (2). Metabolic agents have produced the most provocative recent human signal: glucagon-like peptide-1 receptor agonists, which exert anti-inflammatory and antioxidant effects on the aortic wall, were associated with reduced aneurysm-related events, lower mortality, and fewer repairs in a large observational cohort, independent of diabetes status, motivating prospective trials (37). Targeting the endothelial interface itself, by stabilizing junctions or correcting the cyclic-nucleotide microdomain imbalance through isoform-selective phosphodiesterase modulation, offers a conceptually distinct strategy aimed at the permissive substrate rather than the inflammatory trigger (20). A central caution applies across all of these approaches: because coagulation and innate immunity are physiologically coupled, anti-thrombo-inflammatory therapy must be calibrated to suppress maladaptive amplification without compromising hemostasis, a balance that biomarker-guided patient selection will be essential to strike (10).

Looking ahead, the most plausible clinical path is not a single agent but a stratified, combination approach: matching anti-NET, anti-inflammasome, complement-directed, or barrier-stabilizing therapy to the dominant mechanism in a given patient and disease phase, and reserving systemic anti-inflammatory exposure for those whose molecular and imaging profile marks them as high risk. Repurposing already-approved drugs such as colchicine and GLP-1 receptor agonists lowers the barrier to trials, while the endothelial and cyclic-nucleotide markers discussed earlier could serve as the companion biomarkers that define eligibility and monitor response. In this sense, therapeutic targeting and biomarker development are not separate goals but two halves of the same translational program.

A candid appraisal must also address why this preclinical promise has not yet translated into aortic-specific clinical benefit. Much of the causal evidence for NET, inflammasome, and complement targeting derives from murine models whose aortic biology, hemodynamics, and disease timescale differ substantially from human aneurysm and dissection (4, 6, 9), and favorable effects on wall integrity in mice have repeatedly failed to reproduce as event reduction in patients across cardiovascular disease more broadly. The one large positive anti-inflammatory trial in cardiovascular disease, CANTOS, was not aortic and came at the cost of a significant excess of fatal infection (35), underscoring that suppression of innate immunity carries its own hazard. No completed randomized trial has yet tested an anti-thrombo-inflammatory strategy against hard aortic endpoints, and the physiological coupling of coagulation and innate immunity means that any such therapy must be shown not to compromise hemostasis. These considerations temper, without negating, the therapeutic rationale developed above.

## 9 Thrombo-inflammation as a missing variable in intervention timing

Contemporary guidelines anchor operative thresholds to maximal aortic diameter and growth rate, with class I indications for repair commencing at 5.5 cm for sporadic thoracic aortic aneurysm and at 5.0 cm for high-risk phenotypes including bicuspid aortopathy and heritable connective tissue disorders (1). These thresholds carry clinical utility because diameter is reproducible, universally measurable, and correlates broadly with event risk across population-level analyses. Yet the evidence base that established them also exposed their most consequential limitation: a substantial proportion of acute type A dissections and aortic ruptures occur in patients whose aortic diameter falls below the threshold at which guidelines would recommend prophylactic repair. In the International Registry of Acute Aortic Dissection, more than 60% of type A events occurred in aortas measuring less than 5.5 cm at the time of the acute event (38), a finding independently corroborated across multiple cohorts (39). This epidemiological gap is not a statistical artifact but a biological signal: it reflects the fundamental mismatch between a geometric surrogate and the biological process it is meant to predict.

Diameter captures the cumulative mechanical consequence of wall degeneration but is blind to the rate at which the thrombo-inflammatory program reviewed here is actively dismantling the extracellular matrix, depleting smooth muscle contractile capacity, and destabilizing the endothelial barrier. Two aortas of identical diameter may carry radically different biological risk depending on the degree to which these coupled loops are engaged. A wall that is metabolically active with inflammasome signaling, NET formation, complement deposition, and cyclic-nucleotide-driven barrier fragility is physiologically closer to failure than a geometrically identical wall in which these programs are quiescent. Framing thrombo-inflammation as a candidate missing variable therefore invites us to treat intervention timing as, in part, a biological problem that diameter alone may not fully resolve. We advance this as a hypothesis to be tested rather than as an established determinant of clinical decisions.

The clinical implication is direct. The endothelial signatures, circulating biomarkers, and imaging indices reviewed in Secs. 5 and 6 collectively provide a molecular map of this biological activity. Their integration into a composite risk instrument, validated prospectively against hard endpoints including dissection, rupture, and aortic death, would permit biology-informed triage: identifying the patient at 4.8 cm whose thrombo-inflammatory profile warrants closer surveillance or earlier repair, while providing reassurance in the patient at 5.4 cm whose molecular and metabolic indices are quiescent. This paradigm echoes the transition already achieved in coronary artery disease, where physiological indices including fractional flow reserve now complement angiographic stenosis grading as decision tools (40), and it anticipates a future in which aortic surveillance intervals and repair thresholds are calibrated not to diameter alone but to the biological state of the wall.

## 10 Toward a biology-informed threshold: a conceptual framework

We define a biology-informed threshold as a decision rule for aortic surveillance and intervention timing that augments, rather than replaces, maximal diameter and growth rate (1) with a quantitative estimate of the biological activity of the wall. Four features specify the concept. First, what it is: a composite of anatomic and biological variables in which diameter remains the anchor and biological indices modify the estimated proximity to failure. Second, which biological domains it should incorporate: the thrombo-inflammatory, matrix-remodeling, and endothelial-barrier axes reviewed here, sampled through circulating analytes and, where feasible, through molecular imaging. Third, how these interact with established anatomic parameters: biology is intended to reclassify risk within, not to override, the diameter strata, raising or lowering surveillance intensity and advancing or deferring the timing of repair at the margins where anatomic criteria are ambiguous. Fourth, its intended scope: the concept is developed for prognosis, surveillance, and intervention timing, and explicitly not for primary diagnosis, for which established imaging already suffices.

Operationalizing this vision requires a composite instrument that integrates complementary readouts of the thrombo-inflammatory cascade. A plausible construct would combine a thrombo-inflammatory analyte, such as circulating citrullinated histone H3 (2) or the neutrophil-to-lymphocyte ratio (25), with a matrix-turnover readout, such as circulating MMP-9 or elastin degradation products, an endothelial or wall-derived signature, such as PDE10A (20) or PTPRJ (16) if accessible via liquid biopsy or circulating extracellular vesicles, and a metabolic imaging index such as 18F-fluorodeoxyglucose or 18F-sodium fluoride aortic wall uptake (28), weighted against established anatomic and clinical variables including maximal diameter, growth rate, valve morphology, sex, and heritable risk profile.

Such an instrument would need to satisfy several conditions before entering clinical practice. First, prospective validation in adequately powered, multicenter cohorts with sufficient follow-up to capture hard aortic events. Second, demonstration of incremental predictive value beyond diameter and growth rate alone, using methods such as net reclassification improvement and integrated discrimination improvement. Third, sex-specific and phenotype-specific calibration, given the documented biological differences between bicuspid and tricuspid aortopathy (15) and between thoracic and abdominal disease. Fourth, standardized assay protocols with defined cutoffs and reproducibility data, analogous to the standardization that enabled natriuretic peptides to enter heart failure guidelines (41).

Two points must be stated without qualification to preserve scientific balance. Maximal diameter, together with growth rate, remains the only prospectively validated predictor available to guide the timing of aortic intervention (1); the biomarkers and signatures reviewed here currently generate hypotheses; and no circulating or tissue biomarker has been prospectively validated for surgical timing. It is therefore useful to grade the evidence underlying this framework into three tiers. The first, clinically validated, comprises anatomic diameter and growth rate for intervention (1) and D-dimer for the diagnosis of acute dissection (22). The second, supported chiefly by experimental and animal-model evidence with corroborating human tissue data, comprises the causal roles of neutrophil extracellular traps (2, 4), NLRP3-interleukin-1 beta signaling (5, 6), and complement (9) in wall degradation. The third, exploratory and hypothesis-generating, comprises the endothelial and cyclic-nucleotide transcriptomic signatures derived from single tissue cohorts (16, 18, 20). Locating each mechanism in its proper tier is essential so that biological plausibility is not mistaken for clinical maturity.

A concrete and deliberately illustrative scenario clarifies how such a framework might operate. Consider two patients, each with a 5.2 cm ascending aneurysm below the 5.5 cm operative threshold (1) and each with stable diameter over the preceding year. In the first, circulating markers of thrombo-inflammation and matrix turnover are unremarkable and, where available, metabolic wall imaging is quiescent; a biology-informed reading would support continued imaging surveillance at the standard interval. In the second, the same anatomic picture is accompanied by elevated thrombo-inflammatory and matrix-turnover indices and focal wall uptake on imaging; here a biology-informed reading might justify shortened surveillance intervals or earlier multidisciplinary discussion of repair. This scenario is hypothetical and is offered only to make the concept tangible. It is not a validated algorithm, and no biomarker cutoff currently exists to support such decisions, which remain, for now, governed by diameter and growth rate.

Until such evidence accrues, diameter-based thresholds remain the standard of care and the appropriate reference frame for clinical decisions. The contribution of a thrombo-inflammatory framework at this stage is not to replace them but to expose the biological gap they leave, to provide a conceptually coherent rationale for why that gap exists, and to equip the field with a structured research agenda for closing it. Each molecular axis identified in this review, from the NET-inflammasome-complement triad to the junctional and cyclic-nucleotide endothelial programs, represents both a candidate biomarker node and a potential therapeutic target in the intervention-timing problem that still governs clinical practice.

## 11 Future directions

Three priorities follow from this framework. First, resolution: single-cell and spatial multi-omics are needed to localize the thrombo-inflammatory and endothelial programs to specific cell populations and wall layers, moving beyond the bulk-tissue signatures that currently dominate the field and clarifying, for example, whether transcriptional changes in phosphodiesterase or junctional genes reflect altered cell state or altered cellular composition (20). Second, integration: the inflammatory, remodeling, and endothelial axes should be combined with imaging and clinical variables into composite, computationally derived risk models, so that molecular biology refines rather than merely accompanies the diameter-based decisions that still govern practice. Machine-learning approaches are well suited to synthesizing these heterogeneous data layers into actionable preoperative risk.

Third, therapeutics: the mediators reviewed here are increasingly druggable. Translating any of these will require the biomarker validation described above, so that the right patients can be identified before, rather than after, the wall fails. A thrombo-inflammatory framework, anchored at the endothelial interface, offers a coherent path from molecular driver to clinical strategy, and the field is positioned to translate it into meaningful clinical benefit for patients with aortic dissection and aneurysm.

## 12 Limitations

Several limitations of the evidence base reviewed here warrant explicit acknowledgment. First, much of the mechanistic evidence originates in murine models, whose aortic wall composition, hemodynamic environment, immune repertoire, and disease timescale differ from those of human aneurysm and dissection, so that causal inferences drawn in mice transfer to patients only provisionally. Second, the human data are overwhelmingly observational, derived from cross-sectional case-control comparisons and retrospective cohorts that establish association rather than causation and are vulnerable to confounding and reverse causation. Third, the tissue-based transcriptomic and proteomic studies that underpin the endothelial and biomarker sections are subject to sampling bias, since surgically resected specimens represent advanced disease at a single time point and a single anatomic site and are frequently drawn from single-center cohorts of limited size without independent replication.

Fourth, outcome definitions across the literature are heterogeneous, spanning diameter growth, dissection, rupture, reintervention, and all-cause mortality, which complicates synthesis and comparison. Fifth, and most consequential for the central thesis, there are at present no randomized, biomarker-guided trials in aortic disease and only limited prospective validation of any circulating or tissue marker against hard aortic endpoints. As a narrative rather than systematic review, this synthesis is additionally subject to selection and interpretation bias in the choice and weighting of studies. These limitations do not undermine the biological coherence of the thrombo-inflammatory framework, but they do bound its current clinical applicability and define the prospective, standardized, and endpoint-driven work required before it can inform practice.

## 13 Conclusion

Across its clinical phenotypes, aortic dissection and aneurysm are increasingly understood as expressions of a shared thrombo-inflammatory process in which coagulation and innate immunity actively dismantle the wall rather than merely accompany its structural failure. This review traced that process from its core mediators, neutrophil extracellular traps, the NLRP3 inflammasome, complement, and platelet-leukocyte crosstalk, through the proteolytic and phenotypic remodeling they drive, to the endothelial interface whose junctional, channel, and cyclic-nucleotide integrity determines whether a thrombo-inflammatory insult is contained or converted into wall destruction. The same framework reorganizes the downstream clinical questions, lending mechanistic coherence to circulating and imaging biomarkers, to the inflammatory responses that follow endovascular repair, and to an emerging set of druggable targets. The central hypothesis emerging from this synthesis is that the aorta may fail as a function of how strongly these coupled loops are engaged, and not merely of how dilated it has become, and that progress is likely to follow from biology-informed stratification, validated prospectively and integrated with imaging and clinical data, to complement rather than replace the diameter thresholds that remain the validated standard of care. Realizing that promise will require resolving the cascade at cellular resolution, translating wall-level signals into reliable circulating readouts, and testing targeted anti-thrombo-inflammatory therapy without compromising hemostasis. These remain substantial hurdles, but a coherent path from molecular driver to clinical strategy is beginning to take shape.

## References

[1] Czerny M Grabenwöger M Berger T Aboyans V Della Corte A Chen EP. EACTS/STS guidelines for diagnosing and treating acute and chronic syndromes of the aortic organ. Ann Thorac Surg. (2024) 118(1):5–115. 10.1016/j.athoracsur.2024.01.021 38416090

[2] Zhao Y-F Zuo Z-A Li Z-Y Yuan Y Hong S-C Fu W-G. Integrated multi-omics profiling reveals neutrophil extracellular traps potentiate aortic dissection progression. Nat Commun. (2024) 15(1):10736. 10.1038/s41467-024-55038-8 39737994 PMC11686284

[3] Lyu X Liu Q Shi J Chen Y Dai X. Neutrophil extracellular traps: emerging drivers and therapeutic targets in abdominal aortic aneurysm pathogenesis. Exp Biol Med (Maywood). (2026) 250:10781. 10.3389/ebm.2025.10781 41574096 PMC12822572

[4] Yang M Zhou X Pearce SWA Yang Z Chen Q Niu K. Causal role for neutrophil elastase in thoracic aortic dissection in mice. Arterioscler Thromb Vasc Biol. (2023) 43(10):1900–20. 10.1161/ATVBAHA.123.319281 37589142 PMC10521802

[5] Wu D Ren P Zheng Y Zhang L Xu G Xie W. NLRP3 (nucleotide oligomerization domain-like receptor family, pyrin domain containing 3)-caspase-1 inflammasome degrades contractile proteins: implications for aortic biomechanical dysfunction and aneurysm and dissection formation. Arterioscler Thromb Vasc Biol. (2017) 37(4):694–706. 10.1161/ATVBAHA.116.307648 28153878 PMC5364046

[6] Xiang J He L Xu H. Role of NLRP3/caspase-1/IL-1β inflammasome pathway in formation of aortic dissection in mice. J Army Med Univ. (2024) 46(7):705–14. 10.16016/j.2097-0927.202306105

[7] Takahashi M. NLRP3 inflammasome as a common denominator of atherosclerosis and abdominal aortic aneurysm. Circ J. (2021) 85(12):2129–36. 10.1253/circj.CJ-21-0258 33883388

[8] Wortmann M Skorubskaya E Peters AS Hakimi M Böckler D Dihlmann S. Necrotic cell debris induces a NF-*κ*B-driven inflammasome response in vascular smooth muscle cells derived from abdominal aortic aneurysms (AAA-SMC). Biochem Biophys Res Commun. (2019) 511(2):343–9. 10.1016/j.bbrc.2019.02.051 30782482

[9] Martinez-Pinna R Madrigal-Matute J Tarin C Burillo E Esteban-Salan M Pastor-Vargas C. Proteomic analysis of intraluminal thrombus highlights complement activation in human abdominal aortic aneurysms. Arterioscler Thromb Vasc Biol. (2013) 33(8):2013–20. 10.1161/ATVBAHA.112.301191 23702661

[10] Stark R Massberg S. Interplay between inflammation and thrombosis in cardiovascular pathology. Nat Rev Cardiol. (2021) 18(9):666–82. 10.1038/s41569-021-00552-1 33958774 PMC8100938

[11] Hou N Zhou H Li J Xiong X Deng H Xiong S. Macrophage polarization and metabolic reprogramming in abdominal aortic aneurysm. Immun Inflamm Dis. (2024) 12(11):e1268. 10.1002/iid3.1268 39530309 PMC11555488

[12] Piechota-Polanczyk A Jozkowicz A Nowak W Eilenberg W Neumayer C Malinski T. The abdominal aortic aneurysm and intraluminal thrombus: current concepts of development and treatment. Front Cardiovasc Med. (2015) 2:19. 10.3389/fcvm.2015.00019 26664891 PMC4671358

[13] Boyd AJ. Intraluminal thrombus: innocent bystander or factor in abdominal aortic aneurysm pathogenesis? JVS Vasc Sci. (2021) 2:159–69. 10.1016/j.jvssci.2021.02.001 34617066 PMC8489244

[14] Rojas-Gil I Peña S Saavedra A Ariza F Carbonell JP Gempeler A. A scoping review of consumptive coagulopathy and disseminated intravascular coagulation in aortic aneurysm and aortic dissection. JVS-Vasc Insights. (2026) 4:100355. 10.1016/j.jvsvi.2026.100355

[15] Magouliotis DE Sicouri S Sicouri N Baudo M Cabrucci F Yamashita Y. Epigenetic biomarkers in thoracic aortic aneurysm, dissection, and bicuspid aortopathy: a comprehensive review. Biomolecules. (2025) 15(4):568. 10.3390/biom15040568 40305299 PMC12024610

[16] Magouliotis DE Fergadi MP Christodoulidis G Svokos AA Svokos KA Bareka M. In-depth bioinformatic study of the cadherin 5 interactome in patients with thoracic aortic aneurysm unveils 8 novel biomarkers. Eur J Cardiothorac Surg. (2021) 61(1):11–8. 10.1093/ejcts/ezab338 34293135

[17] Magouliotis DE Arjomandi Rad A Kourliouros A Viviano A Koulouroudias M Salmasi MY. Transcriptomic analysis of tight junction proteins demonstrates the aberrant expression and function of zona occludens 2 (ZO-2) protein in Stanford type A aortic dissection. J Pers Med. (2023) 13(12):1697. 10.3390/jpm13121697 38138924 PMC10744791

[18] Magouliotis DE Sicouri S Rad AA Skoularigis J Giamouzis G Xanthopoulos A. In-depth computational analysis reveals the significant dysregulation of key gap junction proteins (GJPs) driving thoracic aortic aneurysm development. Hellenic J Cardiol. (2025):S1109-9666(25)00001-6. 10.1016/j.hjc.2025.01.001 39800318

[19] Magouliotis DE Sicouri S Ramlawi B Baudo M Yamashita Y Xanthopoulos A. Transcriptomic analysis exploring the role of aquaporins in the pathogenesis of thoracic aortic aneurysm. Ann Thorac Surg. (2026) 121(4):852–63. 10.1016/j.athoracsur.2025.08.015 40886758

[20] Magouliotis DE Sicouri S Androutsopoulou V Baudo M Cabrucci F Zotos P-A. Family-wide dysregulation of phosphodiesterases alters cAMP/cGMP microdomains in thoracic aortic aneurysm. J Cardiovasc Dev Dis. (2026) 13(1):23. 10.3390/jcdd13010023 41590850 PMC12842147

[21] Magouliotis DE Sicouri S Xanthopoulos A Ramlawi B. Why the aortic organ fails longitudinally: a molecular mirror to biomechanical insight. J Thorac Cardiovasc Surg. (2026) 171(2):e33–4. 10.1016/j.jtcvs.2025.07.006 40753503

[22] Suzuki T Distante A Zizza A Trimarchi S Villani M Salerno Uriarte JA. Diagnosis of acute aortic dissection by D-dimer: the International Registry of Acute Aortic Dissection Substudy on Biomarkers (IRAD-Bio) experience. Circulation. (2009) 119(20):2702–7. 10.1161/CIRCULATIONAHA.108.833004 19433758

[23] Cui J- Jing Z- Zhuang S- Qi S- Li L Zhou J- D-dimer as a biomarker for acute aortic dissection: a systematic review and meta-analysis. Medicine (Baltimore). (2015) 94(4):e471. 10.1097/MD.0000000000000471 25634194 PMC4602956

[24] Feng W Wang Q Li C Wu J Kuang J Yang J. Significant prediction of in-hospital major adverse events by D-dimer level in patients with acute type A aortic dissection. Front Cardiovasc Med. (2022) 9:821928. 10.3389/fcvm.2022.821928 35282336 PMC8907574

[25] Li L Wen C Xie L Wu H. Prognostic value of neutrophil-to-lymphocyte ratio in patients with aortic dissection: a systematic review and meta-analysis. Front Cardiovasc Med. (2026) 12:1631314. 10.3389/fcvm.2025.1631314 41584293 PMC12823981

[26] Biros E Moran CS Wang Y Walker PJ Cardinal J Golledge J. microRNA profiling in patients with abdominal aortic aneurysms: the significance of miR-155. Clin Sci (Lond). (2014) 126(11):795–803. 10.1042/CS20130599 24283299

[27] Maegdefessel L Azuma J Tsao PS. MicroRNA-29b regulation of abdominal aortic aneurysm development. Trends Cardiovasc Med. (2014) 24(1):1–6. 10.1016/j.tcm.2013.05.002 23871588 PMC3815986

[28] Jalalzadeh H Indrakusuma R Planken RN Legemate DA Koelemay MJ Balm R. Inflammation as a predictor of abdominal aortic aneurysm growth and rupture: a systematic review of imaging biomarkers. Eur J Vasc Endovasc Surg. (2016) 52(3):333–42. 10.1016/j.ejvs.2016.05.002 27283346

[29] Wu Q He J Li H Xie L Zeng W Lin X. Outcomes of post-implantation syndrome after endovascular repair for Stanford type B aortic dissection. J Vasc Surg. (2024) 79:1326–38. 10.1016/j.jvs.2024.01.200 38286152

[30] Xie L- Lin X- Wu Q- Xie Y- Zhang Z- Qiu Z- Risk prediction and prognostic analysis of post-implantation syndrome after thoracic endovascular aortic repair. Sci Rep. (2024) 14(1):17376. 10.1038/s41598-024-65877-6 39075074 PMC11286741

[31] Voûte MT Bastos Gonçalves FM Van De Luijtgaarden KM Klein Nulent CGA Hoeks SE Stolker RJ. Stent graft composition plays a material role in the postimplantation syndrome. J Vasc Surg. (2012) 56(6):1503–9. 10.1016/j.jvs.2012.06.072 23092643

[32] Halloum N Meyer A-S Wenkel M Dohle D-S Youssef M Dorweiler B. Aortic remodeling after false lumen embolization in aortic dissection. J Clin Med. (2025) 14(3):763. 10.3390/jcm14030763 39941435 PMC11818290

[33] Wu M Zhang L Bao J Zhao Z Lu Q Feng R. Postoperative glucocorticoid enhances recovery after endovascular aortic repair for chronic type B aortic dissection: a single-center experience. BMC Cardiovasc Disord. (2016) 16:59. 10.1186/s12872-016-0234-2 27013022 PMC4807598

[34] Salata K Syed M Hussain MA De Mestral C Greco E Mamdani M. Statins reduce abdominal aortic aneurysm growth, rupture, and perioperative mortality: a systematic review and meta-analysis. J Am Heart Assoc. (2018) 7(19):e008657. 10.1161/JAHA.118.008657 30371297 PMC6404894

[35] Ridker PM Everett BM Thuren T MacFadyen JG Chang WH Ballantyne C. Antiinflammatory therapy with canakinumab for atherosclerotic disease. N Engl J Med. (2017) 377(12):1119–31. 10.1056/NEJMoa1707914 28845751

[36] Low-dose colchicine inhibit abdominal aortic aneurysm growth trial (COIN). Available online at: ClinicalTrials.gov identifier: NCT05361772 (Accessed April 2, 2026).

[37] Ahn H Kaelber DC Hoell N Ambani R Quatromoni JG Khalifeh A. Glucagon-like peptide-1 receptor agonists are associated with reduced abdominal aortic aneurysm-related events. J Vasc Surg. (2026) 83(4):1079–88.e2. 10.1016/j.jvs.2025.11.021 41276093 PMC13395541

[38] Pape LA Tsai TT Isselbacher EM Oh JK O’Gara PT Evangelista A. Aortic diameter ≥5.5 cm is not a good predictor of type A aortic dissection: observations from the International Registry of Acute Aortic Dissection (IRAD). Circulation. (2007) 116(10):1120–7. 10.1161/CIRCULATIONAHA.107.702720 17709637

[39] Elefteriades JA Farkas EA. Thoracic aortic aneurysm clinically pertinent controversies and uncertainties. J Am Coll Cardiol. (2010) 55(9):841–57. 10.1016/j.jacc.2009.08.084 20185035

[40] Tonino PAL De Bruyne B Pijls NHJ Siebert U Ikeno F van `t Veer M. Fractional flow reserve versus angiography for guiding percutaneous coronary intervention. N Engl J Med. (2009) 360(3):213–24. 10.1056/NEJMoa0807611 19144937

[41] McDonagh TA Metra M Adamo M Gardner RS Baumbach A Böhm M. 2021 ESC guidelines for the diagnosis and treatment of acute and chronic heart failure. Eur Heart J. (2021) 42(36):3599–726. 10.1093/eurheartj/ehab368; Erratum in: Eur Heart J. 2021 Dec 21;42(48):4901. doi: 10.1093/eurheartj/ehab670 34447992

